# Post‐translational modifications of Annexin A2 are linked to its association with perinuclear nonpolysomal mRNP complexes

**DOI:** 10.1002/2211-5463.12173

**Published:** 2017-01-17

**Authors:** Ingvild Aukrust, Linn Andersen Rosenberg, Mia Madeleine Ankerud, Vibeke Bertelsen, Hanne Hollås, Jaakko Saraste, Ann Kari Grindheim, Anni Vedeler

**Affiliations:** ^1^ Department of BiomedicineUniversity of BergenNorway; ^2^ Molecular Imaging Centre (MIC)University of BergenNorway; ^3^ Present address: Centre for Medical Genetics and Molecular MedicineHaukeland University HospitalBergenNorway; ^4^ Present address: Department of PathologyOslo University HospitalUniversity of OsloOsloNorway

**Keywords:** Annexin A2, mRNP complexes, post‐translational modification, Ser phosphorylation, sumoylation, ubiquitination

## Abstract

Various post‐translational modifications (PTMs) regulate the localisation and function of the multifunctional protein Annexin A2 (AnxA2). In addition to its various tasks as a cytoskeletal‐ and membrane‐associated protein, AnxA2 can function as a *trans*‐acting protein binding to *cis*‐acting sequences of specific mRNAs. In the present study, we have examined the role of Ser25 phosphorylation in subcellular localisation of AnxA2 and its interaction with mRNP complexes. Subcellular fractionation and confocal microscopy of rat neuroendocrine PC12 cells showed that Ser25‐phosphorylated AnxA2 (pSer25AnxA2) is absent from the nucleus and mainly localised to the perinuclear region, evidently associating with both membranes and cytoskeletal elements. Perinuclear targeting of AnxA2 was abolished by inhibition of protein kinase C activity, which resulted in cortical enrichment of the protein. Although oligo(dT)‐affinity purification of mRNAs revealed that pSer25AnxA2 associates with nonpolysomal, translationally inactive mRNP complexes, it displayed only partial overlap with a marker of P‐bodies. The phosphorylated protein is present as high‐molecular‐mass forms, indicating that it contains additional covalent PTMs, apparently triggered by its Ser25 phosphorylation. The subcellular distributions of these forms clearly differ from the main form of AnxA2 and are also distinct from that of Tyr23‐phosphorylated AnxA2. Immunoprecipitation verified that these high‐molecular‐mass forms are due to ubiquitination and/or sumoylation. Moreover, these results indicate that Ser25 phosphorylation and ubiquitin/SUMO1 conjugation of AnxA2 promote its association with nonpolysomal mRNAs, providing evidence of a possible mechanism to sequester a subpopulation of mRNAs in a translationally inactive and transport competent form at a distinct subcellular localisation.

**Abbreviations**
AnxA2: Annexin A2 protein
ECM: extracellular matrix
HRP: horse radish peroxidase
IP: immunoprecipitate
NE: nuclear envelope
NGF: nerve growth factor
PC12: rat pheochromocytoma cell line
PKC: protein kinase C
pSer25AnxA2: Ser25 phosphorylated AnxA2
PTM: post‐translational modification
pTyr23AnxA2: Tyr23 phosphorylated AnxA2
SUMO: small ubiquitin‐like modifier
Ub: ubiquitin
UTR: untranslated region

As a multifunctional protein, Annexin A2 (AnxA2) is involved in numerous processes including endo‐ and exocytosis, actin dynamics and mRNA transport. It also acts in DNA replication and repair, and most likely also participates in transcription 1, 2, 3, 4, 5, 6, 7. The distinction between the different cellular functions of AnxA2 is regulated by its post‐translational modifications (PTMs), which determine its interaction with different ligands. The expression of AnxA2 is altered in most cancers and its high expression is related to neoangiogenesis and metastasis 8. Therefore, to understand the function of AnxA2 in various cellular contexts, it is important to find out how the protein discriminates between its multiple roles.

AnxA2 is likely to be present in larger protein complexes, whose composition varies depending on their function, localisation and PTMs 5, 6, 9. The N terminus of AnxA2 contains several major sites for PTMs 2, including the phosphorylation sites Ser11 (counting from the first Ser, as Met is removed *in vivo*), Ser25 and Tyr23, which can modify the structural and functional properties of the protein 10, 11, 12, 13, 14, 15. Phosphorylation of Ser25 increases the accessibility of the mRNA‐ and G‐actin‐binding sites of AnxA2 16. The exposure of these sites most likely results from a change in the position of the highly flexible N terminus 16. In addition, Ser25 phosphorylated AnxA2 (pSer25AnxA2) has been implicated in exocytosis 13, 17, 18, 19, 20, macro‐pinosome motility 21, recycling of lipid rafts 22 and the recruitment of protein kinase C (PKC) to phosphoinositide‐4,5‐biphosphate‐rich membrane domains 23. Furthermore, PKC phosphorylation of Ser11 and Ser25 of AnxA2 dissociates the (AnxA2‐S100A10)_2_ tetramer, prevents the Tyr23 phosphorylation and subsequent translocation of AnxA2 to the cell surface, and initiates the degradation of S100A10 24.

A cytoskeleton‐associated pool of AnxA2 is subjected to ubiquitination, indicating that this PTM plays a role in intracellular targeting of AnxA2 and most likely defines its specific function in this compartment 25.

The Ser25 phosphorylation site is not readily accessible to the solvent 16, 26, suggesting that this modification may be preceded by other PTMs or ligand‐binding events. The close proximity of the N and C termini of AnxA2 raise the possibility that this modification may involve the binding of PKC to the 14‐3‐3‐like PKC‐binding site in the very C terminus of the protein 27 and/or its ubiquitination/sumoylation.

AnxA2 has been identified as both a cellular mRNA‐ 28, 29, 30, 31, 32, 33, 34 and a viral RNA‐binding protein 35. Furthermore, its mRNA recognition motif has been identified 5, 32. AnxA2 associates directly with a subpopulation of mRNAs in cytoskeleton‐associated mRNP complexes 28, including its cognate 31 and c‐*myc* 29, 30 mRNAs. In both cases, AnxA2 binds to a ~ 100 nucleotide region in the 3′‐untranslated regions (UTRs) that appears to form a stem‐loop structure 30, 31. The protein has been implicated in mRNA transport, based on its binding to the localisation element present in the c‐*myc* 3′‐UTR 30, which is responsible for the targeting of this mRNA to the perinuclear region 36. Although a number of mRNA‐binding proteins have been identified as components of mRNP complexes, the organisation and regulation of these complexes remain largely enigmatic. As these complexes undergo dynamic compositional changes, their protein–protein interactions are likely to be regulated by PTMs. Interestingly, several proteins involved in the assembly and nuclear export of mRNP complexes are ubiquitinated, indicating that this PTM is related to the mechanisms that regulate the spatio‐temporal dynamics of the maturing mRNP complexes 37.

Here we present new evidence showing that Ser25‐phosphorylated high‐molecular‐mass forms of AnxA2 – which are also ubiquitinated and/or sumoylated – associate with nonpolysomal mRNP complexes that appear to be enriched in the perinuclear region of PC12 cells. Furthermore, inhibition of PKC inhibits the Ser25 phosphorylation of AnxA2 and prevents its localisation to the perinuclear region and results in the enrichment of AnxA2 at the inner cortical region of the plasma membrane.

## Results and discussion

### Subcellular localisation of pSer25AnxA2

**Figure 1:**
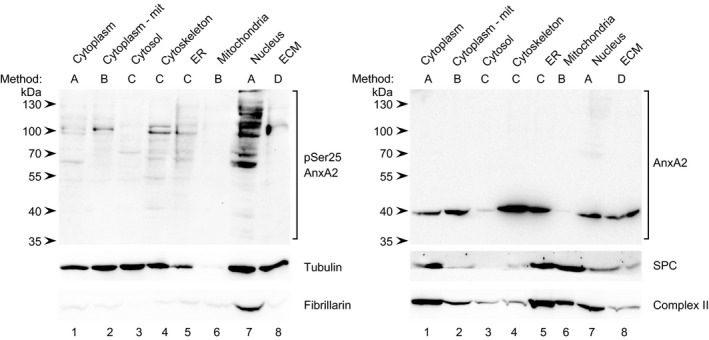
Detection of pSer25AnxA2 in subcellular fractions derived from PC12 cells. Proteins (100 μg) from the cytoplasm (lane 1), the cytoplasm devoid of mitochondria (‐mit; lane 2), the cytosol (lane 3), the cytoskeleton (lane 4), ER (lane 5), mitochondria (lane 6), the nuclear fraction (lane 7) and EGTA‐released ECM (lane 8) were separated by 10% SDS/PAGE and subjected to western blot analysis. The blots were probed with antibodies against pSer25AnxA2 and total AnxA2, as indicated. Antibodies against compartmental markers, namely the cytoplasm (tubulin; 55 kDa), ER (SPC; 25 kDa), nucleus (fibrillarin; 35 kDa) and mitochondria (complex II; 70 kDa) were also employed as indicated. The blot probed against pSer25AnxA2 was reprobed against fibrillarin, while the blot probed against AnxA2 was reprobed against SPC. Only 25 μg of protein from the mitochondrial fraction was used for western blot analysis of tubulin and complex II on two different membranes. Detection of the immunoreactive protein bands was performed using the ChemiDoc™ XRS+ molecular imager after incubation with HRP‐conjugated secondary antibodies and enhanced chemiluminescence (ECL) reagent. The methods (A–D) used to generate the different fractions are indicated above the western blots and described in the Methods section. The arrowheads to the left indicate the protein molecular mass standards.

We previously showed that the phospho‐mimicking AnxA2‐Ser25Glu mutant and AnxA2 Ser25 phosphorylated by PKC are not targeted to the nucleus. Furthermore, the phospho‐mimicking mutant displayed an increased affinity for mRNA *in vitro* 16. To further address the subcellular localisation of pSer25AnxA2 and the functional significance of its ability to bind mRNA, four different methods described in the Methods section were employed to fractionate PC12 cells into the following subfractions (Fig. 1): cytoplasm (lane 1), cytoplasm devoid of mitochondria (lane 2), cytosol (lane 3), cytoskeleton (lane 4), endoplasmic reticulum (ER; lane 5), mitochondria (lane 6), nucleus (lane 7), as well as EGTA‐released extracellular matrix (ECM) proteins (lane 8). Samples from the various fractions were subsequently subjected to 10% SDS/PAGE and western blot analysis (Fig. 1). The present experiments employ a pSer25AnxA2‐specific antibody, which recognises only the native AnxA2‐Ser25Glu mutant, but not the AnxA2‐Ser25Asp mutant. This indicates the specificity of the antibody, as well as supports the recognition of AnxA2‐Ser25Glu as a ‘true’ phospho‐mimicking mutant (Fig. S1). By contrast, the monoclonal antibody against total AnxA2 detects all forms of AnxA2, including the wild‐type AnxA2 and mutated Ser25 variants (Fig. S1), corroborating its use as a general tool. The 39 kDa (36 kDa by SDS/PAGE 2) form of AnxA2 is mainly enriched in the cytoskeletal fraction of PC12 cells (Fig. 1, lane 4), as previously reported for Krebs II, L‐929 and MPC‐11 cells 28. Thus, total AnxA2 is mainly found as a nonmodified 39 kDa monomeric form (Fig. 1). However, longer exposure of the blots rendered the high‐molecular‐mass AnxA2 bands more visible (results not shown), indicating that the high‐molecular‐mass forms of pSer25AnxA2 constitute only a minor fraction of total AnxA2.

According to the results, pSer25AnxA2 is enriched in the nuclear fraction (Fig. 1, lane 7). Moreover, smaller amounts of the protein are present in the cytoskeletal and ER fractions (Fig. 1, lanes 4 and 5 respectively). Thus, the subcellular distributions of pSer25AnxA2 and the main form of AnxA2 are clearly distinct. We expected to find pSer25AnxA2 as a monomer of about 39 kDa. However, Fig. 1 shows that the phosphorylated protein is almost exclusively present in cells as high‐molecular‐mass forms, indicating that it could be subjected to ubiquitination 25 and/or sumoylation, as well 38. As ubiquitination is involved in the association of AnxA2 with the cytoskeleton, it was not surprising to find the phosphorylated, high‐molecular‐mass forms of AnxA2 in the cytoskeletal fraction (Fig. 1, lane 4). However, tubulin is also readily detectable in the nuclear fraction (Fig. 1, lane 7), indicating that this fraction also contains cytoskeletal elements, possibly due to the intimate association of the centrosome with the nuclear envelope (NE) 39.

The enrichment of fibrillarin in the nuclear fraction shows that this fraction is enriched in nucleoplasmic components (Fig. 1). However, the additional presence of tubulin, the signal peptidase complex (SPC) and complex II markers (Fig. 1) indicate that it also contains perinuclear ER membranes, which are in continuity with the NE, as well as the cytoskeletal and/or centrosomal microtubuli and mitochondria. Mitochondria have been shown to closely associate with the NE 40, possibly providing the energy needed for nuclear trafficking. We have previously shown that glyceraldehyde 3‐phosphate dehydrogenase (GAPDH) and topoisomerase solely distribute to the cytoplasmic and nuclear fractions respectively 41.

**Figure 2:**
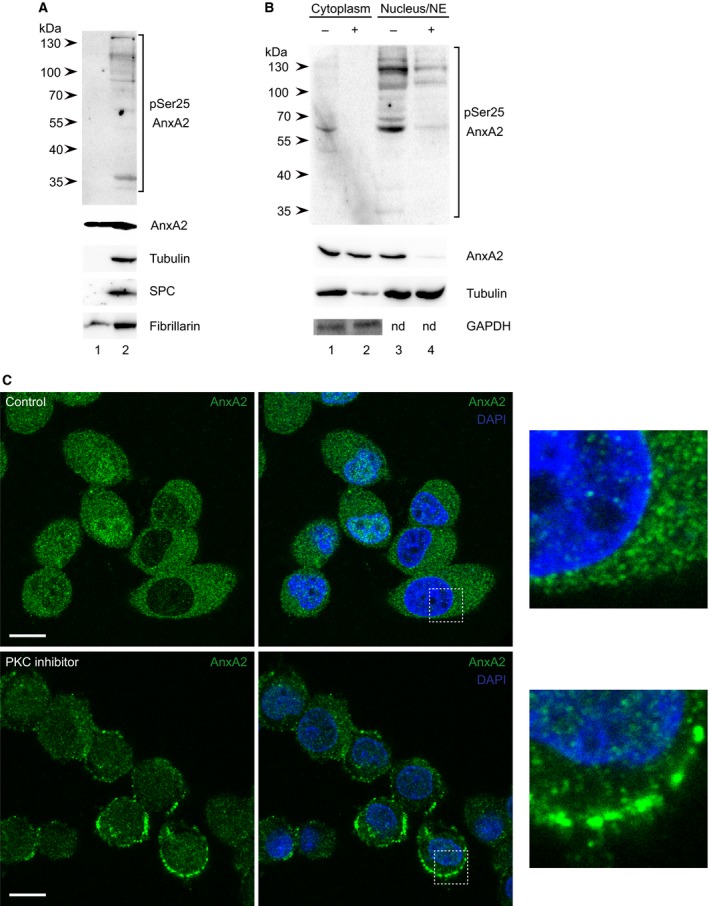
Distribution of pSer25AnxA2 in nuclear subfractions of PC12 cells (A). Proteins (100 μg) from the nucleoplasm (lane 1) and NE (lane 2) fractions were separated by 10% SDS/PAGE and subjected to western blot analysis. The blot was probed with antibodies against pSer25AnxA2, total AnxA2, tubulin, SPC and fibrillarin, as indicated. myr‐ψ‐PKC inhibits Ser25 phosphorylation of AnxA2 and shifts the localisation of AnxA2 from the perinuclear to the cortical region of PC12 cells (B, C). Proteins (100 μg) from the cytoplasmic (lanes 1 and 2) and nuclear (lanes 3 and 4) fractions prepared from myr‐ψ‐PKC‐treated (+; lanes 2 and 4) and control (−; lanes 1 and 3) cells were separated by 10% SDS/PAGE and subjected to western blot analysis (B). The blots were probed with antibodies against pSer25AnxA2, total AnxA2, tubulin and GAPDH, as indicated. Detection of the resulting protein bands (A, B) was performed using the ChemiDoc™ XRS+ molecular imager after incubation with HRP‐conjugated secondary antibodies and ECL reagent. The arrows to the left indicate the protein molecular mass standards (nd, not determined). Immunofluorescence staining of control and myr‐ψ‐PKC‐treated cells, as indicated, was carried out using rabbit polyclonal antibodies against AnxA2 (green) (C). DNA staining with DAPI (blue fluorescence) was included to visualise the nucleus. Scale bar is 10 μm. The insets show higher magnification of the indicated areas in the images shown to the right in Panel (C).

To gain further insight, the nuclear fraction (Fig. 1, lane 7) was further fractionated into the nucleoplasm and the perinuclear membrane fraction, which associates with cytoskeletal proteins/filaments 42. This analysis revealed that pSer25AnxA2 is absent from the nucleoplasm (Fig. 2A, lane 1), but enriched in the membrane fraction (Fig. 2A, lane 2), in agreement with its presence in the ER fraction (Fig. 1, lane 5). Our previous results showing that transfected AnxA2‐Ser25Glu‐GFP and pSer25AnxA2 do not localise to the nucleus 16 corroborate this conclusion. The 39 kDa form of AnxA2 is present both in the nucleoplasm and the NE (Fig. 2A, lanes 1 and 2). Although fibrillarin, a nuclear marker, is detectable in both the nucleoplasmic and membrane fractions, the absence of tubulin and SPC from the former suggests that the subfractionation of the nuclear fraction was successful (Fig. 2A). The presence of fibrillarin in the NE fraction could reflect its shuttling between the nucleus and the cytoplasm 43. Thus, in conclusion, the nuclear fraction includes the NE, perinuclear ER membranes and the nucleoplasm. Furthermore, pSer25AnxA2 is enriched in perinuclear membranes as high‐molecular‐mass forms.

### Effect of PKC inhibitor on the perinuclear localisation of pSer25AnxA2

**Figure 3:**
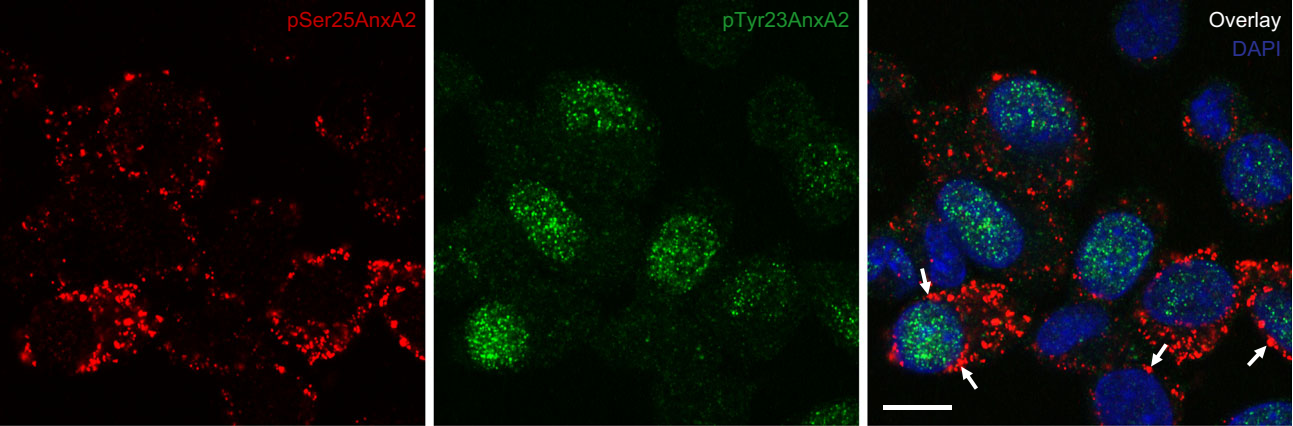
pSer25AnxA2 and pTyr23AnxA2 display distinct subcellular distributions in PC12 cells. Immunofluorescence double‐staining was carried out using rabbit polyclonal antibodies against pSer25AnxA2 (red) and mouse monoclonal antibodies against pTyr23AnxA2 (green), followed by secondary anti‐rabbit and anti‐mouse antibodies coupled to Alexa 594 (red fluorescence) and fluorescein isothiocyanate (FITC) (green fluorescence) respectively. DNA staining with 4′,6‐diamidino‐2‐phenylindole (blue fluorescence) was used to visualise the nucleus. Note that pTyr23AnxA2 (green) is present as a punctate pattern in both the nucleus and the cytoplasm, while pSer25AnxA2 (red) is predominantly found in large punctate structures in the cytoplasm and next to the NE (arrows). Scale bar is 10 μm.

To confirm the role of Ser25 phosphorylation in the generation of the high‐molecular‐mass forms of AnxA2, PC12 cells were treated with myr‐ψ‐PKC, which by inhibiting protein phosphorylation by PKC could influence the level of pSer25AnxA2. As expected, the PKC inhibitor decreased the level of pSer25AnxA2 in the nuclear fraction, which includes the NE and associated perinuclear ER, as well as the cytoplasm (Fig. 2B, compare lanes 3 and 4 and lanes 1 and 2, respectively). Besides indicating that myr‐ψ‐PKC is a highly potent inhibitor of Ser25 phosphorylation of AnxA2, these results also verify that AnxA2 is a PKC substrate 10. The cytoplasmic levels of the 39 kDa form of total AnxA2 in control and PKC inhibitor‐treated (Fig. 2B, lanes 1 and 2 respectively) PC12 cells are very similar. However, the level of the 39 kDa form of total AnxA2 in the nuclear fraction decreases dramatically in response to the inhibitor, while the level of tubulin does not (Fig. 2B, lane 4). Confocal microscopy showed that the PKC inhibitor leads to a cortical enrichment of total AnxA2 at the expense of its presence around the nucleus (Fig. 3C), corroborating the idea that Ser25 phosphorylation is a signal for perinuclear targeting of AnxA2, possibly in the combination with ubiquitination and/or sumoylation. No pSer25AnxA2 could be detected by confocal microscopy when cells had been treated with the PKC inhibitor (results not shown). Inhibition of PKC also decreases the amount of tubulin in the cytoplasmic fraction (Fig. 2B, lane 2), possibly due to a collapse of the microtubule network, or the entire cytoskeleton. Namely, PKC is known to regulate the dynamics of the actin cytoskeleton 44 and PKC‐mediated phosphorylation of α‐tubulin is involved in cell motility and regulation of the length of microtubules 45. By contrast, the level of (GAPDH) in the cytoplasmic fraction was not affected by the treatment (Fig. 2B, compare lanes 1 and 2). In conclusion, inhibition of PKC inhibits perinuclear targeting of AnxA2.

### pSer25AnxA2 and pTyr23AnxA2 show distinct localisations

As subcellular fractionation only provides information about the relative enrichment of specific components, we next employed confocal microscopy to examine the subcellular localisation of pSer25AnxA2. Strikingly, pSer25AnxA2 was localised to punctate structures of variable size around the nucleus. Many of the puncta were found to closely associate with the NE, but many were also localised at a distance from the nucleus (Fig. 3). These results are compatible with our cell fractionation data (Figs 1 and 2), suggesting that pSer25AnxA2 associates with cytoskeletal elements linked to ER membranes that are in continuity with the NE. Dual imaging further showed that Tyr23‐phosphorylated AnxA2 (pTyr23AnxA2) is enriched in the nucleus (Fig. 3), showing that its subcellular distribution differs from that of pSer25AnxA2. Thus, the two phosphorylated forms of AnxA2 are most likely functionally distinct 41. Moreover, these data are in agreement with the finding that the two phosphorylation events at Ser25 and Tyr23 of AnxA2 are mutually exclusive 24. In conclusion, pSer25AnxA2 shows a distinct subcellular localisation different from that of pTyr23AnxA2.

### pSer25AnxA2 associates with perinuclear nonpolysomal mRNP complexes and is ubiquitinated

**Figure 4:**
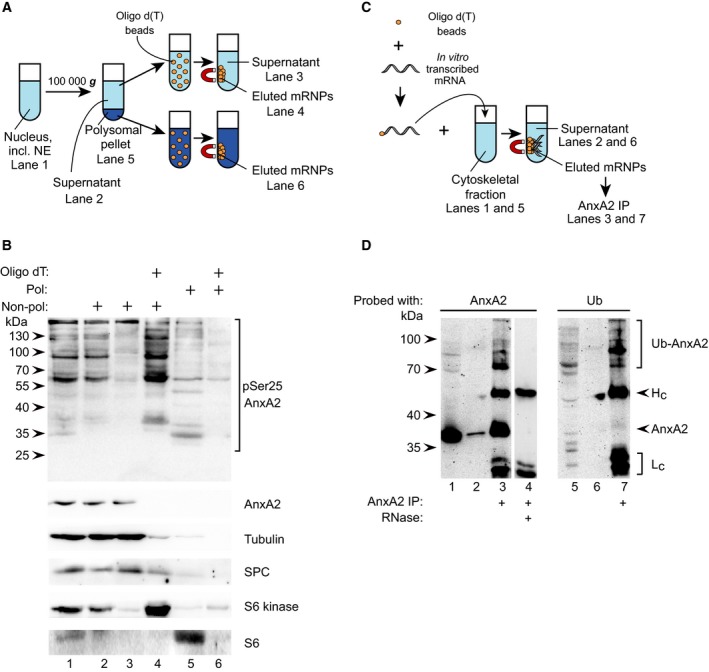
pSer25AnxA2 and ubiquitinated high‐molecular‐mass forms of AnxA2 associate with translationally inactive mRNP complexes. (A, B) High‐molecular‐mass forms of pSer25AnxA2 is present in oligo(dT)‐purified nonpolysomal mRNP complexes in PC12 cells. (A) Schematic representation of the method used in (B) with reference to the individual lanes in (B). (B) Samples (100 μg of protein) were prepared from the following fractions: nucleus (lane 1), supernatant (lane 2) and polysome‐containing pellet (lane 5; derived from the nuclear fraction after centrifugation for 2 h 100 000 ***g*** through a 1 m sucrose cushion), non‐oligo(dT)‐bound supernatant (lane 3), oligo(dT)‐bound supernatant (lane 4), and oligo(dT)‐bound pellet (lane 6), as indicated above the western blot. The proteins were separated by 10% SDS/PAGE and subjected to western blot analysis. The blots were probed with antibodies against pSer25AnxA2, total AnxA2, tubulin, SPC, S6 kinase and the ribosomal protein S6, as indicated. Detection of the resulting protein bands was performed by the ChemiDoc™ XRS+ molecular imager after incubation with HRP‐conjugated secondary antibodies and ECL reagent. The arrowheads to the left indicate the protein molecular mass standards. (C, D) High‐molecular‐mass forms of AnxA2 in mRNP complexes affinity‐purified via binding to *anx*A2 mRNA represent ubiquitinated forms of the protein. (C) Schematic overview of the method used in (D) with reference to the individual lanes in (D). Proteins (100 μg) present in the total cytoskeletal fraction derived from NGF‐stimulated PC12 cells (lanes 1 and 5), the unbound fraction (lanes 2 and 6) and AnxA2 IP proteins from the affinity‐purified mRNP complexes derived from the cytoskeletal fraction (lanes 3 and 7), were subjected to 10% SDS/PAGE and immunoblot analysis using monoclonal antibodies against AnxA2 (lanes 1–4) or Ub (lane 5–7). The bands representing ubiquitinated AnxA2 are indicated by the upper bracket to the right. Lane 4 represents a negative control, showing the binding of cytoskeleton‐associated proteins to *anx*A2 mRNA coupled to oligo(dT) magnetic beads in the presence of RNase, followed by IP using monoclonal AnxA2 antibodies. The molecular mass markers are indicated to the left and the IgG heavy (Hc; arrowhead) and light (Lc; lower bracket) chains to the right.

*In vitro* studies have shown that phosphorylation of Ser25 increases the direct association of AnxA2 with mRNA 16, but the *in vivo* relevance of this finding has not been previously addressed. Therefore, the nuclear fraction (including NE) enriched in pSer25AnxA2 (Fig. 1, lane 7) was further subfractionated into the corresponding polysomal and nonpolysomal populations containing translationally active and inactive mRNAs respectively (Fig. 4A,B). The nuclear polysomal fraction contains no AnxA2 and only negligible amounts of the high‐molecular‐mass bands of pSer25AnxA2 (Fig. 4B, lane 5) associated with poly(A)‐containing mRNAs (Fig. 4B, lane 6), in accordance with previous results showing that the 39 kDa form of AnxA2 associates with cytoskeleton‐bound polysomes 28. This indicates that pSer25AnxA2 is not involved in active mRNA translation in the cytoplasmic (results not shown) or NE‐associated polysomes. By contrast, pSer25AnxA2 and S6 kinase are enriched in the oligo(dT)‐isolated nonpolysomal mRNP complexes, as compared to the starting fraction (Fig. 4B, compare lanes 2 and 4), while S6, a marker of small ribosomal subunits, is enriched in the nuclear polysomal pellet (Fig. 4B, lane 5). This indicates the specificity of the interaction of pSer25AnxA2 with nonpolysomal, NE‐associated mRNP complexes present in the nuclear fraction (Fig. 2). Thus, pSer25AnxA2 could be involved in mRNA transport and/or sequestering of inactive mRNAs in mRNP complexes, most likely in P‐bodies and/or stress granules 46, 47. The finding that AnxA2 binds to the localisation signal in the 3′UTR of c‐*myc* mRNA 30, which targets the mRNA to the perinuclear region for subsequent translation 36, is consistent with this idea.

To show that the high‐molecular‐mass forms of AnxA2 associate not only with the mRNP complexes present in the nuclear fraction but also with specific mRNPs in general, and are ubiquitinated, we used still another approach taking advantage of the fact that AnxA2 binds to its cognate mRNA 31. *In vitro* transcribed and polyadenylated full‐length *anx*A2 mRNA coupled to oligo(dT)‐magnetic beads was used as a ‘bait’ to capture proteins in the cytoskeletal fraction of PC12 cells that had been stimulated with nerve growth factor (NGF) to increase the expression of AnxA2 48. Subsequently, these mRNP complexes were subjected to AnxA2 immunoprecipitation (IP) to recover AnxA2 associated with mRNP complexes (Fig. 4C). Immunoblot analysis of the mRNP complexes isolated by monoclonal antibodies against AnxA2 showed specific enrichment of the high‐molecular‐mass forms of the protein, as compared to the starting cytoskeletal fraction (Fig. 4D, compare lanes 1 and 3). Using monoclonal ubiquitin (Ub) antibodies, the high‐molecular‐mass bands could be identified as ubiquitinated forms of AnxA2 (Fig. 4D, lane 7). Whether ubiquitination targets AnxA2 for proteasomal degradation remains a subject of further studies, as our previous *in vitro* experiments failed to resolve this question 25. The protein has been reported to have a relatively long half‐life (~ 15 h) and to be degraded by chaperone‐mediated autophagy 49, arguing against this possibility.

**Figure 5:**
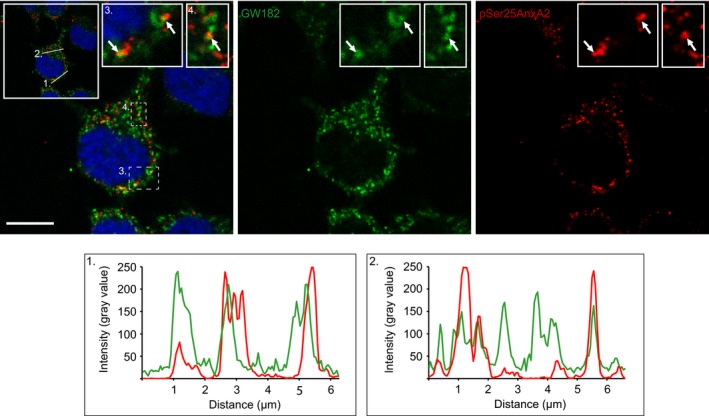
pSer25AnxA2 partially colocalises with the P‐body marker GW182. PC12 cells were double‐stained for immunofluorescence using mono‐ and polyclonal antibodies against GW182 (green) and pSer25AnxA2 (red) respectively. The insets in the merged confocal image to the left – including DAPI staining (blue) to highlight the nuclei – show higher magnifications of the regions, denoted in 3 and 4, to illustrate the partial colocalisation of pSer25AnxA2 and GW182. Scale bar: 10 μm. The fluorescence intensity profiles (from left to right) of the two proteins correspond to the cross‐sections, denoted 1 and 2, shown in the insert in the upper right corner of the merged image.

To gain further insight into the functional role of the pSer25AnxA2‐containing nonpolysomal mRNP complexes, double‐localisation studies with markers of P‐bodies (GW182), stress granules (TIA‐1) and neuronal granules (HuD), which all contain translationally inactive mRNAs, were performed. Of the three marker proteins, pSer25AnxA2 only showed partial colocalisation with the P‐body marker GW182 (Fig. 5, arrows; see also intensity profiles). Arsenite treatment did not increase its colocalisation with any of the markers (data not shown). These studies further suggest that pSer25AnxA2 associates mainly with actively transported mRNP complexes, rather than contributing to the sequestration of the associated mRNAs in or next to P‐bodies, either for transient storage or degradation. Previous results showing that markers of P‐bodies and transported RNPs do not colocalise in the dendrites of mature hippocampal neurons lead to the proposal that dendritic mRNAs could be stored in P‐bodies and subsequently released and translated only after activation of the synapses 50. In conclusion, ubiquitinated and pSer25AnxA2 is a component of nonpolysomal mRNP complexes that, based on confocal microscopy, appear to partially colocalise with P‐bodies.

### High‐molecular‐mass forms of pSer25AnxA2 are modified by Ub and/or SUMO1

**Figure 6:**
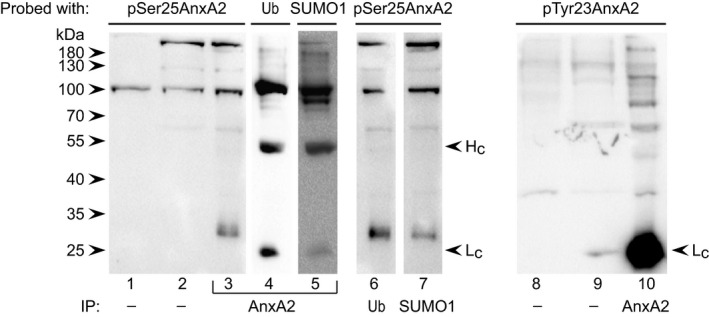
pSer25AnxA2 is ubiquitinated and sumoylated, but shows a different high‐molecular‐mass pattern than pTyr23AnxA2. Proteins (100 μg) in the cytoplasmic (lanes 1 and 8) and nuclear (lanes 2 and 9) fractions (1/6 input) as well as IPs of AnxA2 (lanes 3–5 and 10), Ub (lane 6) or SUMO1 (lane 7) from the nuclear fraction of PC12 cells were subjected to 10% SDS/PAGE and western blot analysis using antibodies against pSer25AnxA2 or pTyr23Anxa2, as indicated. Note that the secondary anti‐mouse HRP‐conjugated antibody obtained from Jackson Immuno‐Research (205‐032‐176) is light chain specific and also note that the blots probed with the polyclonal anti‐pSer25AnxA2 shows no light chain as the antibodies against AnxA2, Ub and SUMO1 are all mouse monoclonal antibodies. The immunoreactive protein bands on the membrane were visualised using ECL reagents.

We showed that pSer25AnxA2 in mRNP complexes is ubiquitinated, but could not rule out that sumoylation is involved, although this modification appears to be particularly relevant for nuclear import 51. Thus, IP of AnxA2, Ub or SUMO1 in the nuclear fraction was performed (Fig. 6). It is evident from the immunoblot that pSer25AnxA2 is both ubiquitinated and sumoylated. There are several examples of proteins that are both ubiquitinated and sumoylated 52. For example, the functions of the PML protein are regulated by phosphorylation, ubiquitination and sumoylation either in combinations or alone (discussed in 52). We previously observed that pTyr23AnxA2 is also ubiquitinated 41, although the pattern of its high‐molecular‐mass forms differs from that of the pSer25AnxA2 (Fig. 6) 41. Furthermore, the two phosphorylated forms of the protein are localised to distinct cellular compartments (Fig. 3). Thus, the post‐translational regulation of AnxA2 is highly complex and may involve cross‐talk between its two termini, as shown for AnxA1 53. Further *in vitro* investigations are hampered by the fact that the E3 ligase for ubiquitination of AnxA2 is unknown. However, it is clear that pSer25AnxA2 shows a more distinct pattern of high‐molecular‐mass forms than pTyr23AnxA2 and appears to be ubiquitinated and/or sumoylated. The ladder of high‐molecular‐mass forms of AnxA2 shown in Fig. 4D, lane 7 is more pronounced than that seen in Fig. 6, lane 4. Namely, in the first case, AnxA2 was immunoprecipitated from purified mRNP complexes formed by its cognate mRNA, while in the latter case, AnxA2 was immunoprecipitated from a subcellular fraction containing not only proteins present in mRNP complexes but also residing in other cellular structures. Overexposure of the blot presented in Fig. 6 revealed the presence of the high‐molecular‐mass forms of Ub‐conjugated AnxA2 (lane 4). Thus, these results support the conclusion that the ubiquitinated (and/or sumoylated forms) of AnxA2 associate with mRNP complexes.

**Figure 7:**
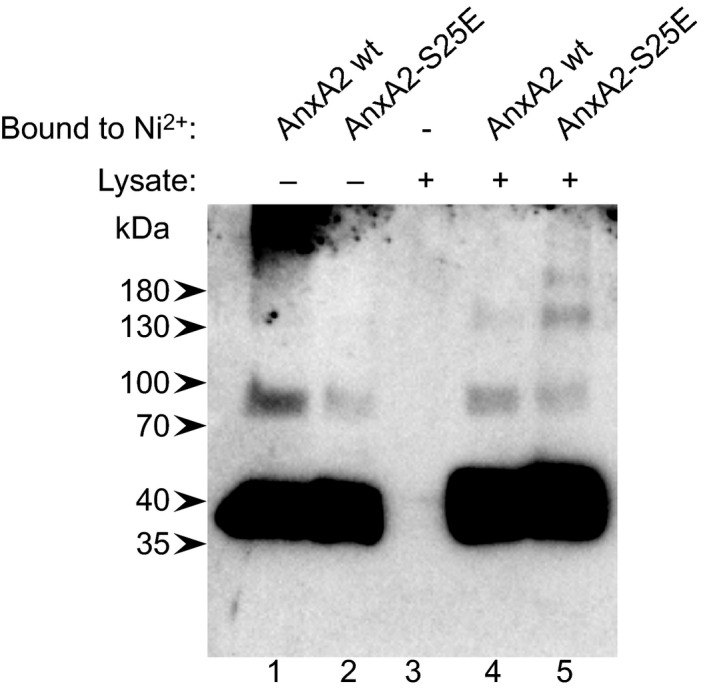
Ser25 phosphorylation appears to trigger the formation of high‐molecular‐mass forms of AnxA2. Recombinant wt His‐AnxA2 (lane 1, 1 μg) and the phospho‐mimicking His‐AnxA2‐Ser25Glu (lane 2, 1 μg) were bound to Ni^2+^‐resin (lanes 4 and 5 respectively) and incubated with PC12 total cell lysate for 1 h before elution with 250 imidazole buffer. 1.5 μg of the eluted wt His‐AnxA2 (lane 4) and His‐AnxA2‐Ser25Glu (lane 5) were loaded on the gel. As a control, elution of proteins present in the PC12 cell lysate that may bind unspecifically to the resin was also performed (lane 3). Proteins were separated by 10% SDS/PAGE and subjected to western blot analysis. The blots were probed with antibodies against AnxA2. Detection of the resulting protein bands was performed using the ChemiDoc™ XRS+ molecular imager after incubation with HRP‐conjugated secondary antibodies and ECL reagent. The arrowheads to the left indicate the protein molecular mass standards.

The presence of AnxA2 in ubiquitinated forms raises the possibility that phosphorylation of Ser25 triggers this modification, as shown for c‐Myc 54. We have not been able to *in vitro* ubiquitinate AnxA2 directly as the required E3 ligase is unknown. To circumvent the problems in using an *in vitro* pSer25AnxA2 in incubations with a lysate containing both kinases and phosphatases, the phospho‐mimicking form of AnxA2 was employed. Thus, experiments where a lysate from PC12 cells was used to *in vitro* ubiquitinate and/or sumoylate recombinant wt AnxA2 and the phospho‐mimicking form, AnxA2‐Ser25Glu, resulted in more high‐molecular‐mass AnxA2 forms after the 1‐h incubation in the latter case (Fig. 7), suggesting that Ser25 phosphorylation could be a trigger for ubiquitination (and/or sumoylation).

Taking into account previous studies implicating ubiquitination in the quality control of nuclear export of mRNAs in yeast 55, together with the finding that AnxA2 binds to the localisation element of the 3′UTR of c‐*myc* mRNA 30, localised to the perinuclear region 36, our present results raise the interesting possibility that ubiquitination (and/or sumoylation) of AnxA2 provides a candidate mechanism to sequester mRNAs in an inactive and transport competent form. This PTM, together with Ser25 phosphorylation of AnxA2 could target these mRNP complexes to specific cellular sites, in particular to the perinuclear region.

## Materials and methods

### Cell culture and drug treatments

Rat pheochromocytoma (PC12) cells derived from adrenal medulla 56 were maintained as described previously 16. For the fractionation of polysomes and mRNP complexes, the cells were treated for 15 min with 100 μg·mL^−1^ cycloheximide (CHX) prior to harvest. Subsequently, the cells were rinsed twice with PBS (0.14 m NaCl, 2.7 mm KCl, 14.5 mm Na_2_HPO_4_, 2.9 mm KH_2_PO_4_) and centrifuged for 5 min at 800 ***g*** before fractionation. Prior to the 30‐min treatment with 100 μm myr‐ψ‐PKC (Promega, Madison, WI, USA), the cells were serum starved for 5 h in a medium containing 1% horse serum and 0.5% fetal calf serum.

### Isolation of subcellular fractions from PC12 cells

A whole PC12 cell lysate was obtained by incubation for 15 min in RIPA buffer (150 mm NaCl, 1% NP‐40, 1% sodium deoxycholate, 0.1% SDS, 25 mm Tris/HCl; pH 7.6) supplemented with 2 mm EGTA and 1× protease inhibitor cocktail (Roche, Mannheim, Germany; EDTA‐free) and centrifuged for 20 min at 12 000 ***g*** at 4 °C. The cytosolic, cytoskeletal and membrane fractions of PC12 cells were isolated essentially as described previously 57 (Method C in Fig. 1). Essentially, PC12 cells from one large flask (7.5 mL of medium supplemented with serum) were lysed for 10 min on ice in 0.1 mL of 25 mm KCl buffer (25 mm KCl, 5 mm MgSO_4_, 8.6% sucrose, 10 mm Triethanolamin; pH 7.4) with 0.075% Triton X‐100, 1× protease inhibitor cocktail (Roche; EDTA‐free) and 200 μm orthovanadate before centrifugation at 800 ***g*** for 10 min. The supernatant contained the cytosol and some released membrane proteins. The pellet was washed once in 25 mm KCl buffer without detergent and centrifuged at 800 ***g*** for 10 min at 4 °C. The resulting pellet was resuspended in 0.1 mL of 130 mm KCl buffer (130 mm KCl, 5 mm MgSO_4_, 8.6% sucrose, 10 mm Triethanolamin; pH 7.4) supplemented with protease inhibitors and orthovanadate. The resuspension was incubated for 20 min at room temperature and centrifuged at 800 ***g*** for 10 min at 4 °C. The supernatant contained the cytoskeleton fraction. The pellet was resuspended in 0.1 mL of 130 mm KCl buffer supplemented with protease inhibitors, orthovanadate and 0.25% Triton X‐100 and 0.25% deoxycholate and incubated for 10 min on ice before centrifugation at 800 ***g*** for 10 min at 4 °C. The supernatant contained membrane‐bound proteins (especially from the ER).

The isolation of cytoplasmic and nuclear fractions was carried out according to the protocol provided in the ‘NE‐PER® Nuclear and Cytoplasmic Extraction Reagents’ kit (ThermoFisher Scientific, Rockford, IL, USA; Method A in Fig. 1). The nuclear fraction was further fractionated into nucleoplasmic and NE fractions using ultracentrifugation in density gradients 58. Polysomes were pelleted by ultracentrifugation for 2 h at 100 000 ***g***_av_ at 4 °C through a 35% (1 m) sucrose cushion prepared in 10 mm Triethanolamine (pH 7.4), 130 mm KCl, 5 mm MgSO_4_ and 70 μm CaCl_2_, essentially as described earlier 59. Nuclear polysomes were released from membranes by the addition of 0.5% Triton X‐100 and 0.5% sodium deoxycholate prior to ultracentrifugation. Poly(A)‐containing mRNAs of mRNP complexes in the resulting supernatant above the sucrose cushion and the polysomal pellet – after splitting of the ribosomes by incubation for 10 min with 50 mm EDTA on ice – were isolated by using of magnetic oligo(dT) Dynabeads, essentially as described by the manufacturer (ThermoFisher Scientific).

Further fractionation of the cytoplasm and harvesting of mitochondria was carried out using the protocol (option A) provided in the ‘Mitochondria Isolation Kit for Cultured Cells’ (ThermoFisher Scientific; Method B in Fig. 1). To obtain a mitochondrial fraction of higher purity, an additional centrifugation step (15 min at 3000 ***g***) was included as a minor modification in the protocol, prior to the collection of the ‘cytosolic fraction without mitochondria’. Furthermore, after the first lysis step, the cells were homogenised with a small grinder to obtain a higher yield of mitochondria.

Extracellular matrix proteins were released by incubation for 10 min in 2 mm EGTA before centrifugation for 5 min at 800 ***g*** (Method D in Fig. 1). Protease inhibitor cocktail (Roche; EDTA‐free) was present during all fractionations.

### *In vitro* transcription and polyadenylation of *anx*A2 mRNA followed by binding to oligo(dT) magnetic beads and binding to cytoskeletal proteins

Bovine *anx*A2 cDNA was subcloned into the pGEM3Zf(+) vector and linearised by *Bam*HI. Subsequently, the full‐length *anx*A2 mRNA containing both UTRs was *in vitro* transcribed as described in the manual for the RibomaxTM Large Scale RNA Production System‐T7 (Promega). Precipitated transcribed mRNA was dissolved in poly‐adenylation (polyA) buffer (50 mm Tris/HCl of pH 7.9, 250 mm NaCl, 10 mm MgCl_2_ and 2.5 mm MnCl_2_), and its purity and integrity was verified by agarose gel electrophoresis. Polyadenylation was performed for 30 min at 37 °C in the presence of 0.03 U·μL^−1^ poly(A) polymerase (Invitrogen; ThermoFisher Scientific, Rockford, IL, USA), 0.5 mg·mL^−1^ bovine serum albumin (BSA) and 0.25 mm ATP. The coupling of poly(A) mRNAs to the oligo(dT) magnetic beads (Dynal; ThermoFisher Scientific, Rockford, IL, USA) was performed essentially as described by the manufacturer, with minor modifications. Accordingly, 50 μg of the poly(A)‐containing *anx*A2 mRNA was diluted in 200 μL binding buffer [20 mm Tris/HCl (pH 7.2), 1 m LiCl, 1.25 mm EDTA], followed by heating for 2 min at 65 °C and subsequent cooling on ice. After washing of the oligo(dT) magnetic beads with binding buffer, the mRNA sample was annealed to the beads in 800 μL binding buffer and incubated for 10 min at RT on a platform shaker, followed by further incubation for 50 min at 4 °C. The *anx*A2 mRNA‐bound oligo(dT) beads were washed extensively with 10 mm Tris/HCl (pH 7.2), 0.15 m LiCl and 1 mm EDTA to remove nonannealed mRNA.

The cytoskeletal fraction (~ 600 μg protein) of PC12 cells was diluted 1 : 4 in RNA‐binding buffer [10 mm Triethanolamine (pH 7.4), 50 mm NaCl, 1 mm DTT, 2 mm MgSO_4_, 1 mm CaCl_2_] containing 1 mg·mL^−1^ yeast tRNA and 0.4 U·μL^−1^ ribonuclease inhibitor (Fermentas, ThermoFisher Scientific, Rockford, IL, USA). Subsequently, the fractions were incubated for 60 min with the *anx*A2 mRNA‐bound oligo(dT) magnetic beads at 4 °C on a platform shaker. The beads were then washed three times with tRNA‐free RNA‐binding buffer. Polyadenylated *anx*A2 mRNAs with bound proteins were eluted from the beads by incubation for 10 min at 65 °C either in 60 μL elution buffer (preheated at 65 °C), containing 0.1% SDS and 1 mm DTT or in 10 mm Tris/HCl (pH 7.4). The elution step was repeated once, and the mRNAs in the two combined fractions were degraded by incubation with 0.3 μg·μL^−1^ RNase A for 5–10 min at 30 °C to facilitate the separation of proteins by SDS/PAGE for immunoblot analyses.

### Protein determination

The protein concentrations of the various subcellular fractions were measured using the Bradford method 60.

### SDS/PAGE, western blot analysis and immunoprecipitation

Proteins were separated by 10% SDS/PAGE and blotted onto nitrocellulose membranes ON at 150 Vh. The membranes were incubated with the different primary antibodies and, subsequently, the corresponding horseradish peroxidase (HRP)‐conjugated secondary antibodies [goat anti‐rabbit (170‐6515; BioRad, Hercules, CA, USA) or goat anti‐mouse (170‐6516; BioRad)] to detect the following proteins in the different fractions: pSer25AnxA2 (OAAF00618; Aviva Systems Biology, San Diego, CA, USA; 1 : 2000 dilution), total AnxA2 (610069; BD Biosciences, San Jose, CA, USA; 1 : 1000 dilution), tubulin (A01410‐40; Genscript, Piscataway, NJ, USA; 1 : 6000 dilution), signal peptidase complex (SPC; 1 : 2000 dilution; a generous gift from S. High, University of Manchester, UK), Complex II (2E3GC12FB2AE2; Invitrogen, ThermoFisher Scientific; 1 : 2000 dilution), fibrillarin (C13C3; Cell Signaling, Danvers, MA, USA; 1 : 2000 dilution), S6 kinase (sc‐8418; Santa Cruz Biotechnology, Dallas, TX, USA; 1 : 100 dilution), S6 (9HCLC; Invitrogen, ThermoFisher Scientific; dilution 1 : 250), and GAPDH (ab9485; Abcam, Cambridge, UK; dilution 1 : 2000), as indicated in the figure legends. Polyclonal AnxA2 antibodies, a generous gift from Jesus Ayala‐Sanmartin (Université Pierre et Marie Curie, Paris, France), were used at a 1 : 5000 dilution. Antibody binding was detected using enhanced chemiluminescence (ECL; Advansta, Menlo Park, CA, USA).

Six hundred microgram of proteins was IP in NET buffer (50 mm Tris/HCl of pH 7.4, 150 mm KCl, 0.05% Triton X‐100, 0.2 mm CaCl_2_ and 2 mm *N*‐ethylmaleimide) using 2.75 μg monoclonal AnxA2 (610069; BD Biosciences), Ub (131600; Invitrogen, ThermoFisher Scientific) or SUMO1 (sc‐5308; Santa Cruz Biotechnology) antibodies coupled to protein G‐Sepharose (Sigma‐Aldrich, St. Louis, MO, USA) essentially as previously described 31. Ribolock (ThermoFisher Scientific) RNase inhibitor was present when using mRNP complexes for IP. Normal mouse IgG (sc‐2025; Santa Cruz Biotechnology) coupled to protein G‐Sepharose was used for preclearance.

### Confocal imaging

PC12 cells were fixed, permeabilised and blocked as described previously 16, 61, prior to staining with primary antibodies against AnxA2 (polyclonal; ab41803; Abcam, 1 : 250 dilution), pTyr23AnxA2 (monoclonal; sc‐135753; Santa Cruz Biotechnologies; 1 : 20 dilution), pSer25AnxA2 (polyclonal; OAAF00618; Aviva Systems Biology; 1 : 250 dilution) and GW182 (monoclonal; sc‐56314; Santa Cruz Biotechnologies; 1 : 10 dilution). The bound primary antibodies were detected using appropriate DyLight 488‐ or DyLight 594‐conjugated goat anti‐rabbit or ‐mouse Fab_2_ fragments (Jackson ImmunoResearch Laboratories, West Grove, PA, USA; 1 : 50 dilution).

## Author contributions

AKG and AV conceived and designed the experiments. IA, LAR, MMA, AKG, VB, HH, IA and AV performed the experiments. IA, MMA, LAR, VB, HH, JS, AKG and AV analysed the data. MMA, LAR, AKG and AV with contributions from JS and IA wrote the paper.

## Supporting information


**Fig. S1.** Specificity of the polyclonal pSer25AnxA2 antibodies and the monoclonal antibody against total AnxA2. [Correction added after online publication on 24 January 2017: Figure S1 replaced with correct version].
