# Supplementary material for: Post‐translational modifications of Annexin A2 are linked to its association with perinuclear nonpolysomal mRNP complexes
Source: FEBS Open Bio. 2017 Jan 17;7(2):160–73. doi: 10.1002/2211-5463.12173 (PMC5292671; doi:10.1002/2211-5463.12173)
Supplement: Supplementary file 1 — Fig. S1. Specificity of the polyclonal pSer25AnxA2 antibodies and the monoclonal antibody against total AnxA2. [Correction added after online publication on 24 January 2017: Figure S1 replaced with correct version]. [file FEB4-7-160-s001.pdf]

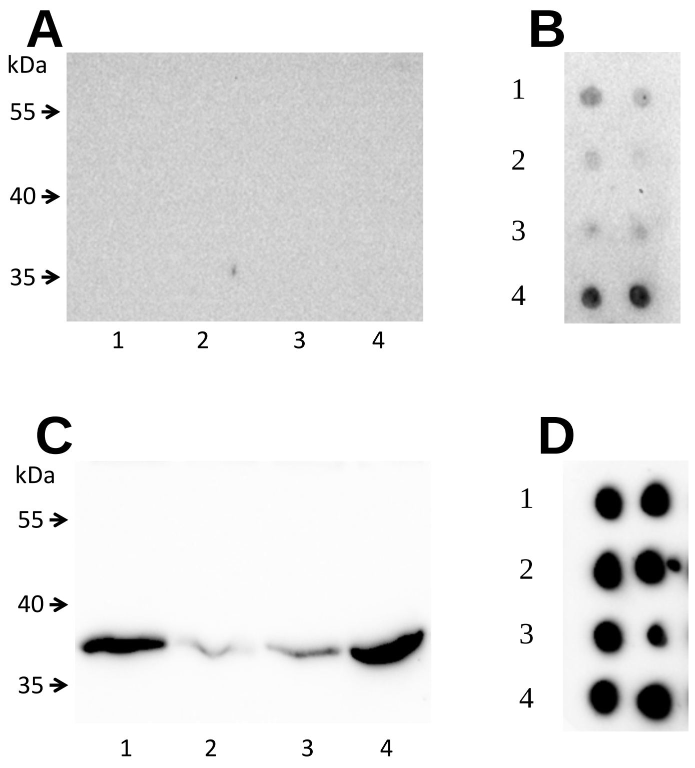

Supplementary Fig. S1. **Specificity of the polyclonal pSer25AnxA2 antibodies and the monoclonal antibody against total AnxA2.** 3 μg (1 μg/μl) of AnxA2 wt (lane 1 and row 1), AnxA2-Ser25Ala (lane 2 and row 2), AnxA2-Ser25Asp (lane 3 and row 3) and AnxA2-Ser25Glu (lane 4 and row 4) were subjected to 10% SDS-PAGE and Western blot analysis (**A** and **C**), and applied on spot blots in their native form (**B** and **D**). The blots were probed with antibodies against pSer25AnxA2 (**A** and **B**) and total AnxA2 (**C** and **D**). Detection of the resulting protein bands was performed using the ChemiDoc^{TM} XRS+ molecular imager after incubation with HRP-conjugated to secondary antibodies and ECL-reagent. The arrows to the left indicate selected protein molecular mass standards. The polyclonal antibodies against pSer25AnxA2 do not recognise denatured AnxA2 wt or any of the denatured Ser25 mutants (Fig. S1A), which indicates the high specificity of these antibodies. However, they do recognise the native form of the AnxA2-Ser25Glu mutant (Fig. S1B, row 4), implying that this mutant represents “a true” Ser25 phospho-mimicking mutant, which justifies its use in confocal imaging. The monoclonal antibody against total AnxA2 recognises denatured and native wt AnxA2 as well as all the tested AnxA2 Ser25 mutants (Fig. S1D and B), especially AnxA2-Ser25Glu (Fig. S1C, lane 4).
